# Microstructure and Mechanical Properties of the 6 wt% Mn-Doped Martensitic Steel Strengthened by Cu/NiAl Nanoparticles

**DOI:** 10.3390/ma16010241

**Published:** 2022-12-27

**Authors:** Yan Jiang, Songsong Xu, Xiuhua Lu, Xiaoxiang Wu, Liang Chen, Shichao Liu, Xinzhong Li

**Affiliations:** 1 School of Iron and Steel, Soochow University, Suzhou 215000, China; 2 Key Laboratory of Neutron Physics, Institute of Nuclear Physics and Chemistry, China Academy of Engineering Physics (CAEP), Mianyang 621999, China

**Keywords:** martensitic steel, nanoparticles, microstructure, mechanical properties, small-angle neutron scattering

## Abstract

The microstructure and mechanical properties of 6 wt.% Mn-doped martensitic steel have been investigated through a combination of electron backscatter diffraction (EBSD), transmission electron microscopy (TEM), and small-angle neutron scattering (SANS). The 6 wt.% Mn-doped steel exhibits a yield strength of ~1.83 GPa and an elongation-to-failure of ~7% under peak aging, and the ~853 MPa of precipitation strengthening is much higher than that observed in the 1.5 wt.% and 3 wt.% Mn-doped steels. The steel is composed of α’-martensite and slightly equiaxed α-ferrite together with a high proportion (~62.3%) of low-angle grain boundaries, and 6 wt.% Mn doping and the aging treatment have an effect on the matrix’s microstructure. However, 6 wt.% Mn doping can obviously increase the mean size of the Cu/NiAl nanoparticles by enhancing the chemical driving force of the Mn partitioning on the NiAl nanoparticles, which differs from the refining effect on the nanoparticles in 3 wt.% Mn-doped steels. Furthermore, larger Cu/NiAl nanoparticles can significantly improve the yield strength of martensitic steel through precipitation-strengthening mechanisms.

## 1. Introduction

High-performance structural steels with high strength, toughness, and ductility are essential for saving energy and enhancing engineering reliability, thus resulting in sustainable economic development. Nanoprecipitation strengthening has been recognized as one of the most effective methods for enhancing the strength of steels [1,2]. For example, Xu et al. developed a new maraging stainless steel-hardening technique by using MC carbides and Ni_3_Ti intermetallic and Cu particles, and they obtained an ultrahigh tensile strength of ~1.6 GPa [3]. Yao et al. found that the precipitation strengthening of nano-sized κ-carbides can effectively increase the yield strength of high-Mn lightweight steel by ~480 MPa and then obtained an outstanding combination of properties, including a tensile strength of 1125 MPa and ~41% total elongation [4]. Raabe et al. developed a new 1.5 GPa TRIP-maraging steel with nanoscale Ni_3_(Ti, Al)-type precipitates. Obviously, the type, number density, size, and spatial distribution of the precipitates, and the interaction of the dislocations with them, decide the strengthening degree in the steels [5]. Among the various types of nanoprecipitates, ultrafine and high-density Cu and NiAl nanoprecipitates constitute two classes of effective strengthening phases for the manufacturing of new low-carbon steels with high strength, high ductility, and good weldability [6,7].

Previous works have mainly focused on the precipitation and growth behavior of Cu and NiAl nanoparticles and their effects on the mechanical properties of Fe-based alloys. In the initial precipitation stage, Cu nanoparticles with a metastable body-centered cubic (BCC) structure and NiAl nanoparticles with an ordered cubic B2 crystal structure were both coherent with the BCC–Fe matrix, and then the Cu nanoparticles gradually transformed into a 9R structure and, finally, a face-centered cubic (FCC) structure [7,8,9]. The precipitation mechanisms of the two nanoparticles were mainly dependent on the Ni/Cu and Al/Cu ratios, which could transfer from Cu prior-precipitation mechanisms to NiAl prior-precipitation mechanisms, seriously influencing the mechanical properties of the steels [10,11]. Mn, as one of the most important elements present in steels, plays a role in the precipitation and growth behaviors of the materials. Jiao et al. found that Mn was apt to partition into NiAl particles by occupying the Al sublattice in 3 wt.% Mn-doped steel, increasing the number density and refining the size of the NiAl nanoparticles, thereby enhancing the precipitation-strengthening effect [12]. Our previous works also found that 9 wt.% Mn doping in medium-Mn dual-phase (DP) steel could induce the formation of Cu/Ni(Mn, Al) precipitates and a higher coarsening coefficient, further influencing the mechanical properties of steels [13]. However, there are obviously different environments for the Cu and NiAl precipitation between Fe-Cu-Ni-Al-Mn-based ferritic steels and medium-Mn DP steels, namely, Cu/NiAl precipitation in medium Mn steels was always accompanied by phase transformation from martensite to austenite, which is controlled by the diffusion of C and Mn.

Under these conditions, it is necessary to exclude the effect of phase transformation on DP steel and understand the role of Mn on the microstructural evolution of the matrix, Cu/NiAl precipitation, and the mechanical properties in the high Mn-doped martensitic steel, furthering the precise regulation of the precipitation strengthening of nanoparticles and obtaining good mechanical properties. Therefore, in this study, 6 wt.% Mn-doped martensitic steel was developed and analyzed regarding its matrix microstructure, the precipitation behavior of the nanoparticles, and its effect on the mechanical properties compared to 1.5 wt.% Mn-doped martensitic steel [14].

## 2. Experimental Procedures

The chemical compositions of the 6 wt.% Mn-doped martensitic steel and the other mentioned steels are presented in Table 1. The steel was fabricated by using vacuum arc melting under an argon atmosphere and then cast into a water-cooled copper mold that used rods with a 20 mm diameter. The ingot was hot-rolled at 1000 °C using multiple passes that ranged from 20 mm to 2 mm, and then the solid solution was treated at 900 °C for 1 h with subsequent water quenching. The resulting material was labeled SS. Finally, the SS samples were aged (AG) isothermally at 500 °C for various periods of time up to 50 h.

Vickers hardness was measured with a load of 1 Kg for 15 s, and ten indents were measured for each specimen to obtain an average value. The tensile strength tests were performed at a strain rate of 10^−3^ s^−1^ using an Instron 5565 testing machine. Tensile specimens with a gage size of 12.5 mm × 1 mm × 4 mm were prepared along the rolling direction, and a 0.2% offset plastic strain method was used to determine the yield strength of the steel.

The electron backscatter diffraction (EBSD) measurements were carried out using a Zeiss Ultra 55 Field-Emission scanning electron microscope (Carl Zeiss Microscopy GmbH, Jena, Germany) (SEM) with an Oxford Nordlys Nano camera (Oxford instruments, Abingdon, UK) and HKL Channel 5 software. X-ray diffraction (XRD) was also applied to identify the phases of the steel. The nanoparticles were characterized by using small-angle neutron scattering (SANS) and transmission electron microscopy (TEM, FEI, Amsterdam, The Netherlands), and SANS was performed at the China Mianyang Research Reactor at the Institute of Nuclear Physics and Chemistry, China Academy of Engineering Physics. Two detector-to-sample distances of 1.8 m and 9 m were applied over a Q range from 0.005 to 0.5 Å^−1^; the details of the characterization and analysis processes can be found in our previous works [13].

## 3. Results and Discussion

**Figure 1:**
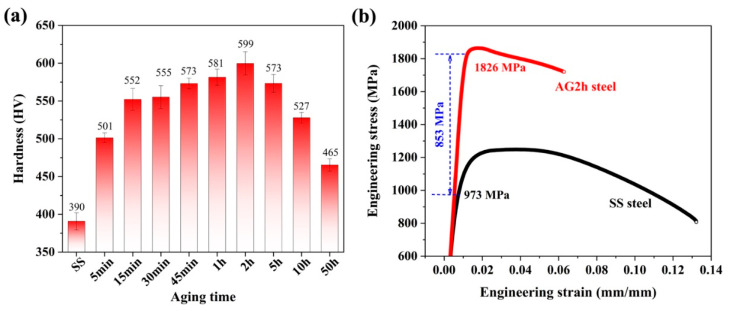
(**a**) Microhardness evolution of the steel aged at 500 °C for different periods. (**b**) Room-temperature tensile stress–strain curves for the SS and AG2h steels.

The microhardness of the 6 wt.% Mn-doped steel under the solid solution (SS) and aged for various lengths of time, and the engineering stress–strain curves of the SS and AG2h steels, is shown in Figure 1. The SS steel has the lowest hardness of ~390 HV. Subsequently, the hardness gradually increases with the increment of aging time, reaching a peak value of ~599 HV after aging for 2 h. Prolonged aging induces the decrement of hardness, i.e., an over-aging effect. The hardness measurements indicate that a substantial age-hardening response formed in the 6 wt.% Mn-doped steel, which was likewise identified with the other Cu/NiAl nanoprecipitate-strengthened steels [15,16]. In contrast, the hardness (~599 HV) of the peak aging in the 6 wt.% Mn-doped steel is higher than that in previous Cu/NiAl-strengthened steels [17,18,19]. Room-temperature tensile tests were performed on the SS and AG2h (i.e., peak aging) steels, and the engineering stress–strain curves are presented in Figure 1b. The SS steel exhibited a yield strength of ~973 MPa, an ultimate tensile strength of ~1249 MPa, and an elongation-to-failure ratio of ~13%. After aging for 2 h, the yield strength increased by ~853 MPa to ~1826 MPa, and the tensile strength increased to ~1863 MPa; furthermore, elongation decreased from ~13% to ~7%. Remarkably, the age-hardening effect after the peak aging time provides ~853 MPa for the yield strength, much higher than that in 1.5 wt.% Mn and 3 wt.% Mn-doped steels [12,14] (shown in Table 2) and the other steels with nanoprecipitation strengthening [20,21,22]. Moreover, the 6 wt.% Mn-doped steel under both under-aging and over-aging conditions had improved elongation-to-facture; however, its strength drastically decreased. In this case, we focused on the mechanical properties of the AG2h steel (i.e., peak aging) to understand the effect of the Mn content on Cu/NiAl precipitation and its strengthening effect. Additionally, the deformation mechanism and fracture behavior will be investigated in detail and published in future work. Thus, the above mechanical properties of the SS and AG2h steels indicate that 6 wt.% Mn doping has an obvious effect on the aging hardening response of martensitic steels.

**Figure 2:**
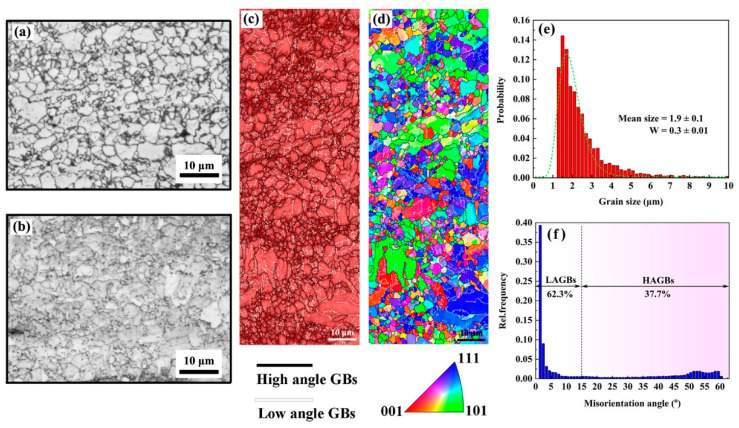
The metallograph of (**a**) SS steel and (**b**) AG2h steel; (**c**) phase map with high and low angle grain boundaries (GBs); and (**d**) inverse pole figure (IPF) map of the SS steel together with (**e**) its grain size and (**f**) grain boundary distributions.

To further understand the effects of the matrix structure, optical microscopy, EBSD, and XRD measurements were carried out on the SS and AG2h steels. The SS steel exhibited a heterogeneous microstructure mainly composed of equiaxed and small grains. The microstructure of the AG2h steel (Figure 2b) is similar to that of the SS steel in terms of both grain morphology and grain size, indicating that the aging treatment has no apparent effect on the microstructure. In Figure 2c,d, most of the body-centered cubic (BCC) structure grains are separated by low-angle grain boundaries (GBs), which are then grouped into martensitic blocks, while only a small portion of equiaxed ferritic grains distribute around the martensite phase. The grain size distribution of the SS steel is presented in Figure 2d, showing that the mean grain size is 1.9 ± 0.1 μm with log-normal function fitting. Notably, the proportion of low-angle GBs (62.3%) in Figure 2f is much more than the high-angle GBs, which are also found in 1.5 wt.% Mn-doped steels, confirming that low-angle GBs evolve from the dislocation walls during the hot-rolled process [14].

**Figure 3:**
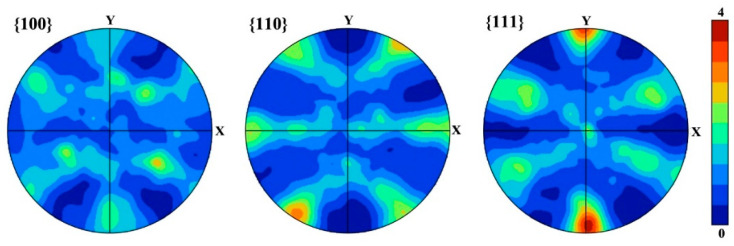
Pole figures for 6 wt.% Mn-doped martensitic steel after solid solution.

**Figure 4:**
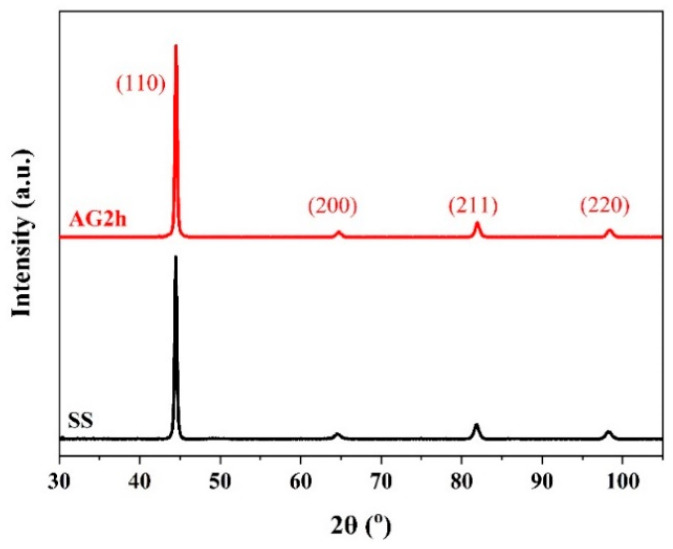
XRD spectrum of the SS and AG2h steels.

The pole figures in Figure 3 show that {111}<110> and {111}<121> textures are formed in the SS steel, and {111}<121> is a recrystallization texture [23], indicating that the SS steel suffers recrystallization during the treatment of the solid solution. The 6 wt.% Mn-doped steel consists of α’-martensite and slightly equiaxed α-ferrite phase together with abundant low-angle GBs, which is in accord with 1.5 wt.% Mn-doped steel [14]. Moreover, the average grain size in the 6 wt.% Mn-doped steel (~1.9 μm) is slightly larger than that in 1.5 wt.% Mn-doped steel (~1.0 μm), as shown in Table 2. The phase information characterized by XRD in Figure 4 shows that both SS and AG2h steels have a single BCC phase, indicating that the aging treatment induces no discernible second phase.

The matrix strengthening in the 1.5 wt.%, 3 wt.%, and 6 wt.% Mn-doped steels mainly comes from the contribution of solid solution strengthening, refinement strengthening, and the work hardening of dislocations. Compared with 1.5 wt.% and 6 wt.% Mn steels, the slight increment in the yield strength of the 1.5 wt.% Mn steel under solid solution conditions (~25 MPa) originates from the refinement strengthening due to its smaller grain size (~1.0 μm) than the 6 wt.% Mn SS steel (1.9 μm), despite the fact that a similar element content except for Mn and the thermomechanical treatments could lead to similar solid-solution strengthening and work hardening in the two steels. However, different chemical compositions and thermomechanical treatments have an obvious effect on matrix strengthening regarding 3 wt.% Mn steel. Therefore, the Mn content has no apparent effect on matrix strengthening under similar chemical compositions and thermomechanical treatments. Additionally, the above three steels have a single BCC phase, verifying that when the content of Mn elements increase from 1.5 wt.% and 3 wt.% to 6 wt.% in the martensitic steel, it has no effect on the matrix phase structure. Moreover, the matrix structure of the 6 wt.% Mn-doped steel underwent no changes after aging for 2 h. Thus, only the precipitation strengthening of the nanoscale precipitates can induce the increment in the strength.

**Figure 5:**
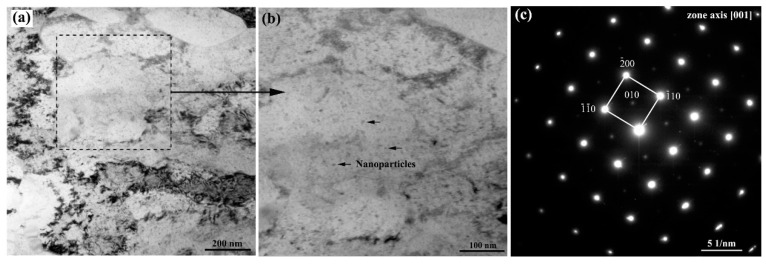
(**a**,**b**) TEM bright-field micrographs for the AG50h steel and (**c**) the respective [001] zone-axis selected area diffraction patterns for the steel with (010)-type superlattice reflections.

TEM and SANS were used to examine the nanoscale precipitates. The bright-field TEM micrographs for the AG50h steel and the respective [001] zone-axis selected area diffraction pattern are shown in Figure 5. It can be seen that many spherical nanoparticles with small radii (<10 nm) were detected in the matrix. The selected area diffraction pattern of the AG50h steel in Figure 1c shows (100)-type superlattice reflections, indicating the existence of a B2-type ordered phase. According to the nanoprecipitation-strengthened steel with similar chemical compositions, the superlattice reflections originate from B2-NiAl nanoparticles [24,25]. Moreover, the Cu and NiAl nanoparticles in the Fe-Cu-Ni-Al-Mn-based steels are invariably precipitated together, forming a coprecipitation of Cu/NiAl [10,26]. Therefore, the above TEM results indicate that Cu and NiAl nanoparticles are formed in the 6 wt.% Mn-doped steels after the aging treatments.

**Figure 6:**
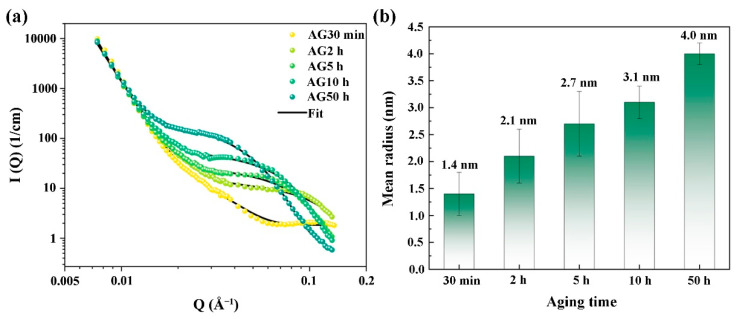
(**a**) SANS-intensity distribution as a function of momentum transfer Q for the steels aged at 500 °C for various times and (**b**) the mean radius of nanoparticles.

Due to their extremely small size and the fact they contain multiple elements, the Cu and NiAl nanoparticles before the over-aging are hard to characterize using TEM. Therefore, the SANS characterization technique is frequently used to research precipitation in alloys, owing to the fact that it enables the observation of the nanoscale precipitates in the materials [27,28]. The SANS data and satisfactory model fitting results are presented in Figure 6. The SANS data are modeled as the sum of $I\left(Q\right)=I_{low}\left(Q\right)+I_{high}\left(Q\right)$, in which $I_{low}\left(Q\right)$ represents the grain boundaries’ scattering with Porod’s power-law scattering, while $I_{high}\left(Q\right)$, which is based on the polydisperse log-normal size distribution, describes the nanoscale precipitates. The details of the fitting can be found in our previous work [29]. As a result of lacking the accurate composition of the nanoparticles in the 6 wt.% Mn-doped steel, only the mean radii of the nanoparticles determined from the fitting are shown in Figure 6b. With increased aging time, the nanoparticles gradually evolve, and the average size increases from ~1.4 nm of the AG30min steel to ~4.0 nm of the AG50h steel. Notably, the nanoparticles’ mean radii in the AG2h steel of the 6 wt.% Mn-doped steel (~2.1 nm, peak aging) are greater than that in the 1.5 wt.% Mn-doped steel (~1.6 nm) and 3 wt.% Mn-doped steel (~1.38 nm) after aging for 2 h [12,14], indicating that the higher Mn content promotes the growth of the nanoparticles. Jiao et al. [12] found that 3 wt.% Mn doping Fe-5Ni-1Al-based steel can increase the number density and refine the size of NiAl nanoparticles due to the higher nucleated rate of the NiAl nanoparticles, which is induced by the significant decrement in the strain energy for NiAl nanoparticle nucleation and the increments in the chemical driving force. However, 6 wt.% Mn doping in the steel obviously increases the mean size of the nanoparticles, and their average radius in the AG2h steel (peak aging) is similar to the 9 wt.% Mn-doped medium-Mn steels under the peak aging conditions (~2.1 nm) in our previous work [13], indicating that the high Mn content in the martensitic steel can markedly enhance the chemical driving force of its diffusion and promote Mn partitioning in the NiAl nanoparticles, further increasing the size of the Cu/NiAl nanoparticles. Therefore, Mn doping ferritic or martensitic steels can refine the radii of the NiAl nanoparticles by decreasing the critical energy needed for their formation as well as increase their size by increasing the chemical driving force of the Mn partitioning to NiAl. Moreover, the refining or coarsening effects on the NiAl nanoparticles in the steels are highly dependent on the Mn content.

Cu and NiAl nanoparticles effectively enhance the strength of martensitic steel by using precipitation-strengthening effects. For the AG2h steel, the radii of the nanoparticles (~2.1 nm) are smaller than the critical radius for the bypass mechanism; therefore, the gilding dislocation would shear the nanoparticles in the steel [30]. The contribution of the shearing mechanism to strength mainly comes from order strengthening, modulus strengthening, chemical strengthening, and coherency strengthening [22]. Among the above four mechanisms, order strengthening and modulus strengthening play a significant role in contributing to yield strength [14]. Additionally, the degree of order strengthening is directly proportional to the radii of the nanoparticles. The larger size of the Cu/NiAl nanoparticles in the AG2h steel compared to 1.5 wt.% Mn and 3 wt.% Mn-doped steel, under the same aging treatment, offers higher precipitation strengthening effects for the AG2h steel (as shown in Table 2 and Table 3). Therefore, 6 wt.% Mn doping martensitic steel can control the precipitation strengthening effect by changing the size of the Cu/NiAl nanoparticles, further influencing the mechanical properties of the steel.

## 4. Conclusions

Based on the systematic research on the microstructure and mechanical properties of the 6 wt.% Mn-doped steel enhanced using Cu/NiAl nanoparticles, the following conclusions can be drawn:

1. The 6 wt.% Mn doping in the martensitic steel has no effect on the matrix microstructure when compared with 1.5 wt.% Mn-doped steel. The 6 wt.% Mn-doped steel is composed of BCC α’-martensite and slightly equiaxed α-ferrite phase together with abundant low-angle GBs, and an aging treatment at 500 °C has no effect on the matrix’s microstructure.
2. The 6 wt.% Mn-doped steel after aging for 2 h (i.e., peak aging) exhibits a yield strength of ~1.83 GPa and an elongation-to-failure ratio of ~7%, and the precipitation-strengthening effect contributes ~853 MPa to its yield strength, which is much higher than that observed in previous Cu/NiAl-strengthened steels.
3. Unlike the refining effect for the nanoparticles with the 3 wt.% Mn doping, the 6 wt.% Mn-doped steel has larger Cu/NiAl nanoparticles than those in the 1.5 wt.% and 3 wt.% Mn-doped steels under the same aging treatment, and the coarsening effect for the nanoparticles is mainly due to the higher chemical driving force of the Mn partitioning to NiAl during its growth stage. Furthermore, the coarse nanoparticles in the 6 wt.% Mn-doped steel by precipitation strengthening effect obviously improve the yield strength of the steel.

## Figures and Tables

**Table 1:** The main chemical compositions for Cu/NiAl nanoprecipitation and grain size of the steels in this study and the referred works.

	Mn	Ni	Cu	Al	Mo	W	Ti	Nb	Si	C	Fe	Refs.
1.5 Mn steel	1.5	4	2.5	1	1.5	1.5	0.1	0.05	0.5	0.08	Bal.	[12]
3 Mn steel	3	5	–	1	–	–	–	–	–	–	–	[14]
6 Mn steel	6	4	2.5	1	1.5	1.5	0.1	0.05	0.5	0.08	Bal.	This work
9 Mn steel	9	4	2.5	1	1.5	1.5	0.1	0.05	0.5	0.08	Bal.	[13]

**Table 2:** Mechanical properties of the nanoprecipitate-strengthened steels with different Mn contents.

	Martrix Microstructure	Yield Strength (MPa)		Strength Increment (MPa)	Grain Size (μm)	Refs.
		Solid Solution	Peak Aging			
1.5 Mn steel	BCC	998	1731	733	1.0	[12]
3 Mn steel	BCC	685	1225	540	13	[14]
6 Mn steel	BCC	973	1826	853	1.9	This work
9 Mn steel	BCC and FCC	619	1269	650	1.0	[13]

**Table 3:** Mean radius of nanoparticles in the 1.5 wt.%, 3 wt.%, 6 wt.%, and 9 wt.% Mn-doped steels with various aging treatment.

	Temperature (°C)	Mean Radius (nm)					Refs.
		30 min	2 h	5 h	10 h	50 h	
1.5 Mn steel	500	1.1	1.6	2.4 (Peak aging)	2.6	3.5	[12]
3 Mn steel	550	–	1.38 (Peak aging)	–	–	–	[14]
6 Mn steel	500	1.4	2.1 (Peak aging)	2.7	3.1	4.0	This work
9 Mn steel	500	2.1 (Peak aging)	-	-	-	-	[13]

## Data Availability

The data presented in this study are available on request from the corresponding author.
